# Supplementary figures and images for: A Feasibility Study for Immediate Histological Assessment of Various Skin Biopsies Using Ex Vivo Confocal Laser Scanning Microscopy
Source: Diagnostics (Basel). 2022 Dec 2;12(12):3030. doi: 10.3390/diagnostics12123030 (PMC9777122; doi:10.3390/diagnostics12123030)

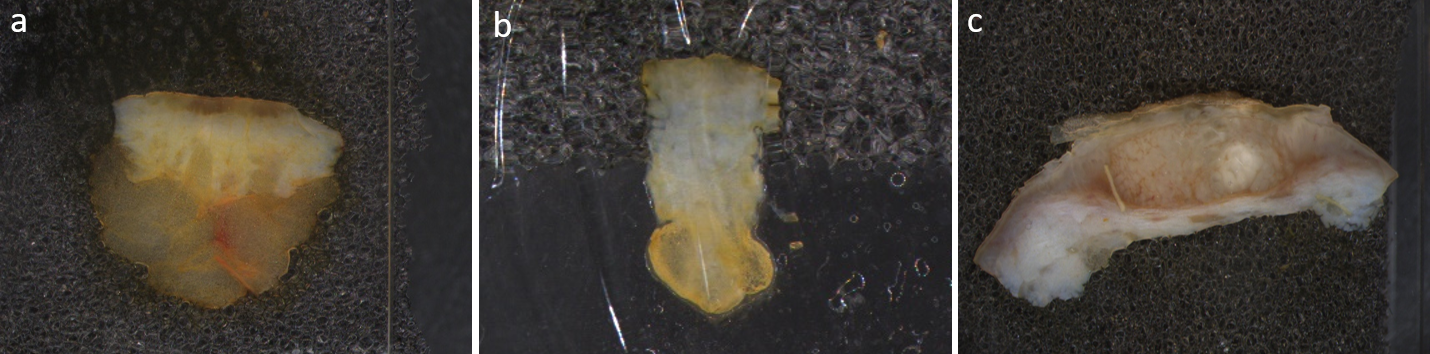

Supplement: Supplementary file 1 [file diagnostics-12-03030-s001.zip › Supplement Figure S1.tif]

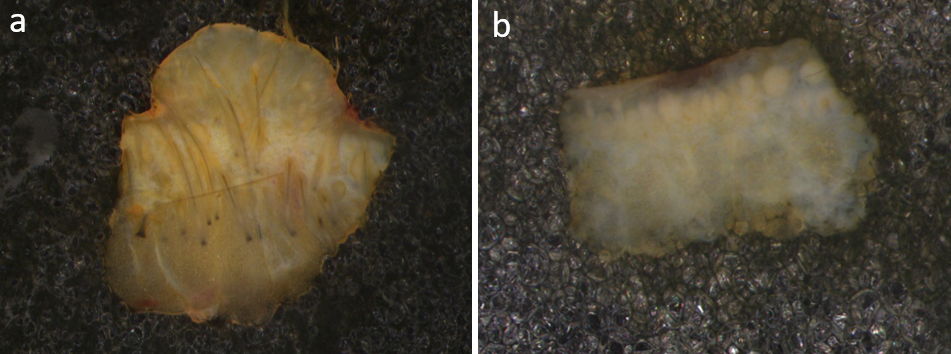

Supplement: Supplementary file 1 [file diagnostics-12-03030-s001.zip › Supplement Figure S2.tif]

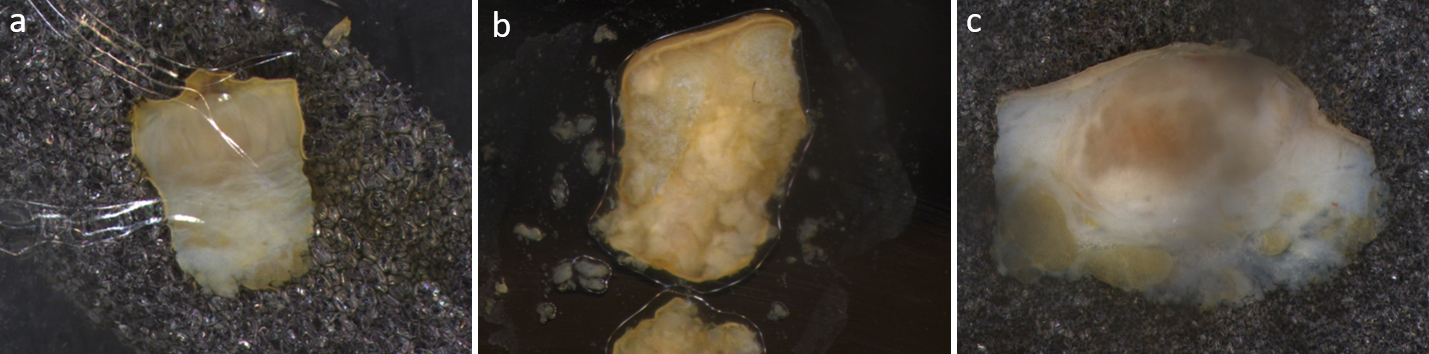

Supplement: Supplementary file 1 [file diagnostics-12-03030-s001.zip › Supplement Figure S3.tif]
